# Low-Cost Sensors in 5G RF-EMF Exposure Monitoring: Validity and Challenges

**DOI:** 10.3390/s26020533

**Published:** 2026-01-13

**Authors:** Phoka C. Rathebe, Mota Kholopo

**Affiliations:** Department of Environmental Health, Faculty of Health Sciences, Doornfontein Campus, University of Johannesburg, P.O. Box 524, Johannesburg 2006, South Africa; motaliok@gmail.com

**Keywords:** 5G RF-EMF monitoring, low-cost sensors, citizen science, calibration uncertainty, participatory monitoring

## Abstract

The deployment of 5G networks has transformed the landscape of radiofrequency electromagnetic field (RF-EMF) exposure patterns, shifting from high-power macro base stations to dense networks of small, beamforming cells. This review critically assesses the validity, challenges, and research gaps of low-cost RF-EMF sensors used for 5G exposure monitoring. An analysis of over 60 studies covering Sub-6 GHz and emerging mmWave systems shows that well-calibrated sensors can achieve measurement deviations of ±3–6 dB compared to professional instruments like the Narda SRM-3006, with long-term calibration drift less than 0.5 dB per month and RMS reproducibility around 5%. Typical outdoor 5G FR1 exposure levels range from 0.01 to 0.5 W/m^2^ near small cells, while personal device use can cause transient exposures 10–30 dB higher. Although mmWave (24–100 GHz) and Wi-Fi 7/8 (~60 GHz) are underrepresented due to antenna and component limitations, Sub-6 GHz sensing platforms, including software-defined radio (SDR)-based and triaxial isotropic designs, provide sufficient sensitivity for both citizen and institutional monitoring. Major challenges involve calibration drift, frequency band gaps, data interoperability, and ethical management of participatory networks. Addressing these issues through standardized calibration protocols, machine learning-assisted drift correction, and open data frameworks will allow affordable sensors to complement professional monitoring, improve spatial coverage, and enhance public transparency in 5G RF-EMF exposure governance.

## 1. Introduction

The fifth generation of mobile communication technology (5G) has been rolled out rapidly worldwide. Since 2018, governments and telecommunication companies have promoted it as essential infrastructure for the digital age, enabling ultra-fast data transfer, low latency, and the connectivity needed for applications like autonomous vehicles, telemedicine, and the Internet of Things (IoT) [1]. However, this vision is based on a physical reality quite different from earlier generations: instead of a few large towers, 5G depends on a dense network of small-cell transmitters embedded in everyday environments, from lampposts and traffic lights to the sides of residential buildings [2,3]. For many citizens, the technology has become unavoidable, not just as a service but as an infrastructure woven into the fabric of urban life. As in past generations, this visibility has been met with unease. The introduction of 2G in the 1990s raised fears about brain tumors from mobile phones; 3G deployment triggered local protests over mast siting near schools; and 4G reignited debates over cumulative exposure and ecological effects [4]. With 5G, however, public concern has intersected with a new information landscape. Social media platforms have amplified both misinformation and distrust, giving unprecedented reach to those questioning the independence of regulators or the adequacy of safety standards [5]. In this environment, fear cannot simply be explained away as ignorance. It reflects a deeper dissonance between institutional framing of safety and community perception of risk.

Regulators like the International Commission on Non-Ionizing Radiation Protection (ICNIRP) and IEEE define safety in terms of numbers: meeting exposure limits set to prevent immediate thermal effects [1]. However, communities view risk more subjectively, considering long-term uncertainty, precautionary ethics, and fairness in how infrastructure is allocated [6]. Independent researchers share this skepticism, highlighting the absence of long-term epidemiological studies and the need for a precautionary approach [7]. As a result, deploying 5G has become more than just a technical project, it is also a test of trust, transparency, and democratic accountability [8]. One of the clearest signs of this trust gap is how exposure is monitored. Professional RF-EMF monitoring efforts use high-quality equipment, but their coverage is limited. Measurements are costly, sporadic, and usually limited to a few sites in major cities [9]. While technically precise, these efforts cannot capture how exposure varies over time, space, and different usage patterns. Even in countries with advanced monitoring systems, large areas remain unmeasured, and because these campaigns are episodic, their data quickly become outdated. Another major issue is accessibility. The results are often hidden in technical reports that are released months or years after the measurements are taken or presented in formats that are hard for non-experts to understand [10]. In countries like Poland, where stricter exposure limits show how uneasy people are [11], this lack of transparency makes it seem like monitoring is just for compliance, not for the public to know. The irony is clear; the infrastructure is on every street, but reliable data about exposure is still mostly invisible to the public.

Low-cost sensors present a viable path forward. Although less accurate than professional equipment, these devices can be widely deployed, providing continuous and distributed data [9,12]. Citizen science initiatives in other fields offer strong examples: PurpleAir’s crowdsourced air quality maps now compete with official monitoring in detail [13]; community radiation mapping projects after the Fukushima disaster offered real-time transparency in ways government systems could not [14,15]; and noise mapping efforts have transformed urban planning debates [16,17]. In telecommunications, grassroots projects in the 2000s mapped Wi-Fi and 4G coverage, foreshadowing the potential of citizen-led exposure monitoring. Today, various initiatives are emerging around RF-EMF. In Greece, the National Observatory of Electromagnetic Fields has developed a network of frequency-selective sensors, some designed to be accessible to the public, providing near-real-time maps of exposure levels [18]. In France, civic groups have distributed affordable meters to households worried about antenna placements, creating a participatory dataset that complements official monitoring efforts [19]. These initiatives demonstrate both the potential benefits and the challenges of citizen-led monitoring. While it can democratize data access, concerns about calibration, reliability, and its acceptance in regulatory processes still persist [13,20,21]. Importantly, democratization involves more than just measurement; it also focuses on equity. Professional monitoring usually gathers in well-funded urban areas, whereas affordable sensors enable rural or marginalized communities to gather and analyze data independently. This process of measurement becomes a political act, giving citizens the opportunity to challenge official accounts and take part in influencing the environment they are exposed to [22,23,24]. Recent empirical evaluations of affordable 5G RF-EMF sensors offer both promising insights and clear limitations. For example, a distributed Sub-6 Ghz sensor network in Belgium and the Netherlands [9] experienced an average calibration drift of about 0.4 dB per month during outdoor use. Laboratory tests of SDR-based prototypes showed deviations of 1.78 dB under controlled conditions and up to 5.26 dB in field tests when compared to the professional Narda SRM-3006 m [25]. Similarly, a triaxial 5G sensor by Van der Straeten et al. [26] achieved a mean deviation of 2.8 dB within the reference equipment’s uncertainty margin, demonstrating that low-cost sensors can attain measurement accuracy within ±5 dB. However, quantitative assessments for mmWave (>24 GHz) bands are still largely lacking, with most research focusing on Sub-6 GHz frequencies. This highlights the need for comprehensive reviews that not only cover sensor development but also evaluate their calibration reliability, qualitative performance, and spatial coverage potential within participatory 5G monitoring frameworks.

This paper contends that inexpensive sensors offer a vital chance to bridge the gap between institutional assurances and public doubts regarding 5G RF-EMF exposure monitoring. By increasing coverage, fostering citizen involvement, and enhancing transparency, they can shift exposure monitoring from an exclusive, technocratic task to a more inclusive democratic process. However, this potential is not guaranteed. Without careful focus on validity, interpretation, and governance, low-cost monitoring may generate data that regulators dismiss or that could be exploited in misinformation efforts. Therefore, the stakes go beyond technical concerns; they also involve the legitimacy of institutions, equitable access to information, and the future of public trust in digital infrastructure governance. Recent studies have made notable progress in validating low-cost 5G RF-EMF sensors, including SDR-based prototypes [25], triaxial 5G exposure sensors [26], and reviews of assessment technologies [27]. However, these works mainly concentrate on device calibration and technical performance. This review complements that by offering a broader, interdisciplinary overview that combines metrological, methodological, and societal perspectives of low-cost sensing. It places technical findings into the context of citizen science involvement, data interoperability, and policy frameworks, such as the European SEAWAVE and GOLIAT projects. The key contribution of this paper is providing a comprehensive view of how affordable sensing technologies can enhance transparency, inclusivity, and standardization in 5G RF-EMF exposure monitoring, bridging the gap between technical validation and governance.

## 2. Methodology

This review critically examines the emerging role of low-cost sensors in monitoring RF-EMF exposure from 5G networks, highlighting their technical validity, deployment challenges, and research gaps. For this review, “low-cost” refers to devices priced under approximately $1000 USD, in contrast to professional-grade meters such as the Narda SRM-3006, which cost over $10,000. This difference reflects roughly a tenfold cost gap often used in recent RF-EMF and environmental sensing research [9,25], separating affordable, community-level sensors from high precision professional equipment. It examines both commercially available sensors and academic prototypes, including those based on software-defined radios (SDRs). The review mainly concentrates on technologies validated for sub-6 GHz 5G exposure monitoring, while also exploring emerging research into the mmWave (24–100 GHz) range. Since no single low-cost platform currently covers both frequency ranges, the study emphasizes existing developments and identifies mmWave sensing as a significant research gap. The scope encompasses technical performance, methodological issues, and the broader societal implications of using these tools. The literature search was conducted using Scopus, IEEE Xplore, PubMed, Web of Science, and Google Scholar, using keywords like “5G RF-EMF exposure,” “low-cost sensors,” “mmWave sensing,” “beamforming measurement,” and “citizen science EMF.” Gray literature, including documents from ICNIRP, WHO, national regulators, and NGO-led initiatives, was also reviewed. This review was updated to include studies published through 2025, ensuring coverage of the most recent advances in low-cost and hybrid rf-emf exposure assessment, including analyses of broadband and personal exposimeter systems [28]. The focus was on publications from 2015 onward, aligning with the start of 5G infrastructure deployment. The inclusion criteria encompassed studies reporting empirical RF-EMF exposure data collected with low-cost or consumer-grade sensors.

Along with 5G New Radio emissions, the reviewed literature also examined overlapping Wi-Fi technologies (IEEE 802.11 a/b/g/n/ac/ax/be and ah, corresponding to Wi-Fi 4–8 and HaLow), which primarily operate within the 2.4 GHz, 5 GHz, 6 GHz, and 60 GHz bands. These frequency ranges significantly overlap with Sub-6 GHz 5G allocations (3.3–4.2 GHz), making them detectable by most broadband or SDR-based low-cost sensors used in this study. Although Halow (802.11 ah, 0.9 GHz) and Wi-Fi 7/8 (6–60 GHz) extend beyond the sensors’ calibrated ranges, their emissions can still influence overall broadband readings. To manage this overlap, the studies included sensors that were either frequently calibrated or colocated with reference equipment to differentiate Wi-Fi traffic from 5G downlink signals. Temporal sampling was also considered. Since Wi-Fi and 5G signals are bursty and load-dependent, exposure levels can fluctuate rapidly over milliseconds, whereas international standards average exposure over 6 min. Most affordable sensors record field strength at 1–10 Hz and employ moving averages or root-mean-square calculations to align with these reference intervals. Some prototypes buffer data or synchronize with network activity to detect transient peaks while maintaining averaged compliance measurements [9,25]. These design methods enable low-cost sensors to adapt to RF device duty cycles and estimate real-time exposure levels within an acceptable uncertainty range (usually ±3–6 dB).

Theoretical studies on propagation that did not involve physical measurements were excluded. Additionally, health-related research was considered only if it evaluated exposure levels using low-cost data. For each selected study or initiative, technical details were gathered, including sensor type, frequency range, calibration methods, measurement uncertainties, and development context. These details helped categorize common challenges into three groups: metrological issues like calibration drift and limited frequency coverage; data quality concerns, such as lack of standardization and interpretability; and gaps in implementation and scalability. The analytical framework used critical synthesis and triangulation to identify convergences, such as widespread calibration difficulties, and divergences, especially in how different applications interpret and handle uncertainty. While aiming for comprehensive coverage, the study has several limitations. Rapid advancements in 5G and sensor technologies mean some challenges noted here might soon become obsolete. Additionally, there are few rigorous assessments of low-cost sensors operating in mmWave bands, leaving important gaps. Lastly, the literature tends to focus on promising prototypes, which may underrepresent failed or inconclusive efforts. This methodology offers a structured foundation for evaluating the validity and relevance of low-cost 5G RF-EMF sensing technologies in both scientific research and societal applications.

## 3. 5G and RF-EMF Fundamentals

### 3.1. Key Technological Features of 5G

The fifth generation of mobile networks (5G) represents a major redesign of wireless communication systems. It is not just a single technology, but a suite of innovations aimed at providing extremely high data speeds, low latency, and vast device connectivity. Unlike earlier generations, 5G uses two primary frequency ranges: Sub-6 GHz and millimeter-wave (mmWave) bands. The Sub-6 GHz spectrum, usually from 700 MHz to 3.8 GHz, improves on 4G’s coverage and capacity. Meanwhile, mmWave frequencies (24–100 GHz) support multi-gigabit data rates but have shorter transmission distances and are more easily blocked by obstacles such as foliage and buildings [27]. To address this propagation issue, 5G utilizes Massive-Input Multiple-Output (MIMO) antenna arrays and beamforming, which actively direct radio beams towards users to optimize signal strength and spectral efficiency [29]. Features like carrier aggregation, which merges multiple frequency blocks to increase throughput, and Dynamic Spectrum Sharing (DSS), enabling the concurrent use of 4G and 5G on the same frequencies, further improve network versatility. These advances considerably change the exposure landscape. Transitioning from a few high-power macrocells to a dense network of low-power small cells creates highly variable, dynamic electromagnetic environments. Power densities fluctuate rapidly due to traffic, user movement, and beam steering. While this network densification is essential for meeting 5G performance targets, it also presents new challenges in measuring exposure, developing models, and effectively communicating with the public [30].

### 3.2. Principles of RF-EMF Exposure

International exposure guidelines for RF-EMF are primarily established by the International Commission on Non-Ionizing Radiation Protection [31] and the IEEE International Commission on Electromagnetic Safety [32]. Both organizations set exposure limits based on the lowest scientifically validated threshold for adverse health effects, determined from experimental and epidemiological studies, rather than solely focusing on preventing immediate thermal responses. In these frameworks, fundamental basic restriction is expressed as the whole-body-averaged Specific Absorption Rate (SAR), measured in watts per kilogram (W/kg). SAR indicates the energy absorption rate from electromagnetic fields by body tissues. For far-field exposures, the more practical reference levels, electric field strength (V/m) and power density (V/m^2^), are derived from SAR limits through computational and experimental methods using anatomically realistic human models [33]. These conversions include conservative reduction factors to account for biological variability among individuals and uncertainties in modeling, ensuring that meeting the reference levels also ensures compliance with the SAR safety thresholds. The WHO’s EMF Project coordinates efforts to review scientific evidence and promote global consistency in exposure assessment methods [34,35]. Currently, international safety standards recognize thermal effects as the only scientifically proven health impacts of RF-EMF exposure below regulatory limits [31,36]. While some research groups explore potential non-thermal biological effects, such as oxidative stress or DNA alterations, these results are inconsistent and not considered confirmed health risks by the ICNIRP, IEEE, or WHO. The ICNIRP Data Gap Report highlights these topics for further study but affirms that current exposure limits are sufficiently protective [37]. Nevertheless, ongoing public disclosure and independent reviews, like the EUROPAEM EMF Guidelines and the BioInitiative Working Group, support the need for continued scrutiny of long-term, low-intensity exposure [38,39]. These differing perspectives highlight the dual challenge for policymakers: to uphold traceable, evidence-based standards while transparently responding to evolving scientific knowledge and community concerns. Developing a credible and balanced regulatory framework will depend on sustained interdisciplinary dialogue and openness to new methodologies.

### 3.3. Existing Exposure Assessment Methods

Accurate RF-EMF exposure assessment relies on reliable measurement tools and standardized methods. Common professional instruments include isotropic E-field probes, spectrum analyzers, and frequency selective meters. Isotropic probes measure fields uniformly along three orthogonal axes, providing orientation-independent readings. Spectrum analyzers offer frequency-resolved data, enabling differentiation between technologies like Wi-Fi, 4G, and 5G New Radio (NR) [40]. Field assessments usually use one of three methodological approaches: driven-by surveys, stationary monitoring stations, and personal dosimeters [29]. Driven-by surveys involve mobile campaigns using equipment mounted on vehicles to capture spatial variations. These are effective for city-scale mapping but are limited to accessible routes and only provide temporal snapshots [41]. Stationary monitoring stations provide continuous long-term measurements at fixed locations, offering high temporal resolution but limited spatial coverage. Greece’s National Observatory of Electromagnetic Fields exemplifies this with its nationwide 5G-inclusive network, enabling real-time public reporting [18]. Personal dosimeters are lightweight wearable devices that track individual exposure in daily life. They are vital for epidemiological research, but face calibration difficulties and biases caused by body shielding [29]. Recent research highlights the need for measurement techniques to evolve with the growing technical complexity of 5G. Beamforming, quick power changes, and dynamic spectrum management render traditional averaging insufficient [27]. Additionally, enclosed and reflective settings like vehicles and buildings create standing-wave patterns, which complicate standard probe calibration [42]. Although professional instrumentation remains the standard for accuracy, its cost and logistical requirements limit spatial and temporal coverage. As a result, researchers and regulators increasingly acknowledge the complementary role of low-cost, networked sensors to enhance official monitoring systems. Properly integrating these distributed data streams through calibration, metadata standards, and transparent governance provides a pathway towards more comprehensive and participatory exposure assessment within the 5G environment.

Table 1 compares professional, low-cost, and participatory approaches to RF-EMF exposure monitoring. It highlights a trade-off between accuracy and accessibility: professional systems offer precision but limited transparency and coverage, while citizen and low-cost methods increase reach and participation at the cost of standardization. Bridging these models is crucial for establishing a more credible and democratic framework for 5G exposure assessment. Beyond methodological differences, exposure characteristics vary notably between user equipment (such as smartphones, tablets, and IoT wearables) and mobile infrastructure (for example, base stations and small cells). Measurements taken in public environments typically show that ambient RF-EMF from mobile networks contributes only a small portion of total personal exposure, usually between 5 and 20% of the total dose. User devices tend to dominate short-term peaks, especially during uplink data transmissions. For example, Velghe et al. [29] observed that exposure from a user’s own device can be 10–30 dB higher than environmental levels, depending on usage patterns and proximity. Similarly, Remirez-Vazquez et al. [41] reported that base-station power densities in dense urban areas average 0.01–0.1 W/m^2^, while localized exposures from handheld devices can transiently reach 1–10 W/m^2^ at close proximity to the body. These differences highlight the importance of distinguishing between various sectors—residential, occupational, transportation, and medical—and between user-side and network-side sources when interpreting or validating low-cost sensor data. This distinction ensures that environmental field measurements are not confused with personal device emissions, thereby enhancing scientific accuracy and public communication.

## 4. The Landscape of Low-Cost RF-EMF Sensors

### 4.1. Commercial Solutions

**Figure 1:**
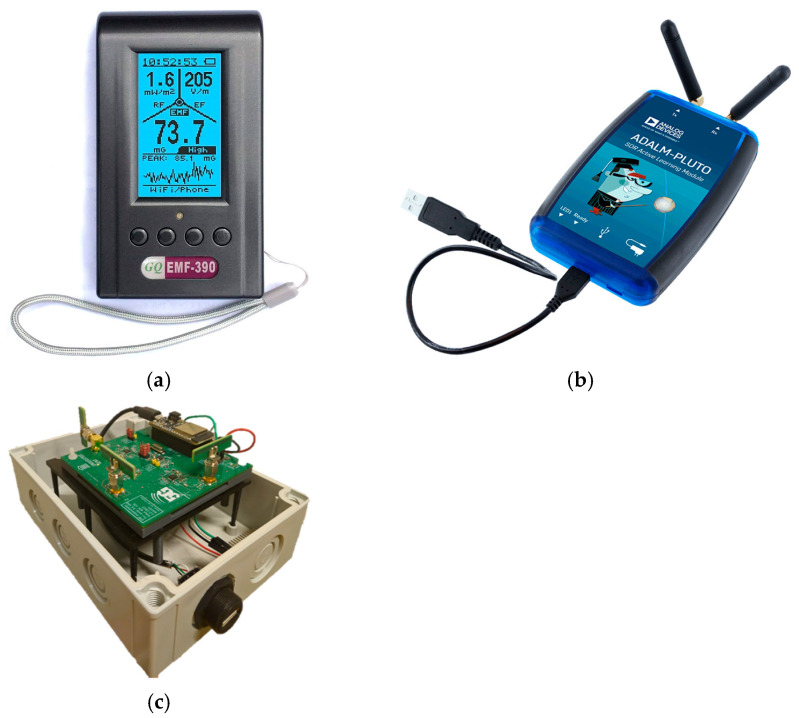
Representative Low-Cost and Research-Grade 5G RF-EMF Sensors. Examples of (**a**) GQ EMF-390 handheld meter (open access image), (**b**) Adla-Pluto SDR platform (image courtesy of manufacturer website and open access) and (**c**) Triaxial 5G RF-EMF research sensor [26].

In recent years, a small but expanding market of low-cost RF-EMF sensors has emerged to supplement professional monitoring systems. These commercial options, often aimed at hobbyists or small organizations, offer affordable, continuous exposure measurement with minimal expertise needed [43]. Examples include portable handheld meters, low-power IoT sensor nodes, and reconfigured SDRs. Table 2 lists the main specifications of selected off-the-shelf sensors. These devices generally work across Sub-6 GHz frequencies and sometimes cover the 3.5 GHz 5G NR band (Further examples in Figure 1a–c). Their dynamic range is usually limited (typically 0.1–20 V/m), and their measurement uncertainty tends to be higher than that of professional isotropic probes, generally ±3–6 dB. While some manufacturers claim frequency selectivity, most sensors employ broadband detection, which makes source attribution more challenging. Prices range from under 300 euros for basic handheld meters to 1500–2000 euros for SDR-based multi-band detectors [25].

While these commercial sensors can track overall exposure trends, their accuracy is still significantly lower than laboratory-grade equipment. Validation studies show average deviations of 2.5 to 5 dB when compared to professional monitors like the Narda SRM-3006, a difference that is often within the measurement uncertainty of the reference device [26].

### 4.2. Academic and Open-Source Prototypes

Alongside commercial innovation, academic and open-source communities have created various research prototypes that use SDRs, IoT chips, and inexpensive triaxial antennas [49,50]. These designs seek to balance cost-effectiveness with scientific openness. For example, researchers from Ghent University and The Hague University of Applied Sciences compared SDR-based sensors, such as the Adalm Pluto, with institutional prototypes like imec-WAVES and Smart Sensor Systems devices. They observed average deviations of only 1.78 dB in laboratory conditions and 5.26 dB in outdoor environments [25]. More recently, a low-cost triaxial 5G sensor designed to measure the n77 and n78 bands (3.3–4.2 GHz) showed a sensitivity of 0.06 V/m and an uncertainty of 3.12 dB, with an average deviation of just 2.8 dB compared to the SRM-3006 reference. This indicates a promising potential for large-scale deployment [26]. The open-source approach of many academic designs enables community validation and continuous improvement. Platforms like GitHub (v. 2.52.0) provide shared firmware, calibration scripts, and data repositories, promoting transparency that proprietary systems lack. This openness aligns with trends in citizen sensing areas like air quality and noise monitoring, where sharing calibration methods and ensuring reproducibility are key to building trust [51].

### 4.3. Citizen Science Initiatives

Low-cost sensors have also sparked a new wave of citizen science projects aimed at mapping RF exposure in daily environments. These initiatives build on the tradition of participatory mapping that was established earlier with Wi-Fi and 4G networks. An early example is the RF-EMF exposure sensing network in Antwerp, which used eleven fixed and five mobile low-cost sensors to track local exposure patterns. The system generated high-resolution spatial and temporal data, validated with professional equipment, demonstrating the viability of city-wide participatory EMF monitoring [12]. Similar projects have also emerged in France and Greece, where national observatories provide publicly accessible dashboards that combine official and citizen-generated data [18]. Citizen projects not only produce data but also build trust and encourage engagement, providing the public with a tangible way to understand and discuss exposure. Nonetheless, lessons from related environmental sensing fields reveal ongoing challenges: uneven data quality, inconsistent calibration, and unrealistic expectations of empowerment without proper data governance [52]. Achieving sustainable success requires consistent calibration, thorough metadata documentation, and ongoing feedback loops between citizens, scientists, and regulators.

### 4.4. Benchmarking Against Professional Equipment

Comparative validation is essential for confirming the reliability of low-cost sensors. Benchmarks conducted in labs and real-world settings regularly show accuracy differences that are significant but manageable. In a 2023 multi-lab comparison, low-cost 5G sensors differed from professional probes by an average of 1.78 dB and up to 5.8 dB in field tests [25]. Another validation in 2025 found deviations within 2.8 dB, which is within the uncertainty range of the commercial reference device [26]. Comparative validations offer specific insights into the quantitative performance of low-cost 5G sensors. Dprez et al. [25] compared four SDR-based prototypes and found mean deviations of 1.78 dB in controlled laboratory conditions and up to 5.26 dB in outdoor field trials, chiefly due to temperature drift and multipath interference. Similarly, Van der Straeten et al. [26] validated a triaxial 5G sensor (3.3–4.2 GHz) against the Narda SRM-3006 reference, observing an average deviation of 2.8 dB, well within the reference uncertainty (±3 dB). Aerts et al. [9] reported that in a distributed RF-EMF sensor network of 35 nodes, long-term calibration drift reached approximately 0.4 dB per month when uncorrected, highlighting the need for periodic co-location checks. These examples demonstrate that, when properly calibrated, Sub-6 GHz low-cost sensors can maintain total uncertainty below ±5 dB, which is acceptable for environmental monitoring though not yet sufficient for regulatory compliance. Although low-cost sensors show promising results, they face challenges such as signal drift, temperature sensitivity, and calibration decay over time [26,53,54]. Without frequent recalibration or reference checks, measurement uncertainty can exceed 5–7 dB, affecting comparability. Additionally, their frequency selectivity is limited: Sub-6 GHz sensors are becoming more reliable, but mmWave bands (>24 GHz) remain mostly out of reach for low-cost devices due to limitations in antennas and components [27]. When properly calibrated and transparently documented, low-cost sensors offer useful supplementary data to professional systems. They are especially valuable for detecting spatial exposure gradients, temporal changes, and public risk perceptions. Recent studies have extended this benchmarking work to incorporate intelligent mapping and hybrid measurement frameworks. For example, Najera et al. [28] compared broadband sensors and personal exposimeters using evolutionary programming optimization to develop high-resolution EMF exposure maps. Their results indicated that hybrid calibration between static broadband sensors and wearable exposimeters reduced spatial error by approximately 25% compared to standalone devices. Such research illustrates how algorithmic correction and data fusion can compensate for inherent uncertainties in low-cost sensors, aligning well with the discussion on machine-learning-based calibration in Section 5.1. Similarly, ongoing projects in Spain and Belgium demonstrate how integrating software intelligence into sensor networks can significantly enhance spatial accuracy and data usability. The way ahead is not about replacing reference-grade equipment but about integrating citizen-generated and institutional data within a well-organized, quality-controlled monitoring framework.

## 5. Core Challenges in Low-Cost 5G Sensing

### 5.1. Metrological and Technical Hurdles

Despite notable progress, inexpensive 5G RF-EMF sensors still encounter key metrological challenges that affect their long-term reliability and regulatory approval. The primary concern is calibration precision and the impact of temporal drift [25]. While initially calibrated against reference instruments, many sensors experience calibration loss over time caused by temperature changes, component aging, and electromagnetic interference [53,55,56]. Laboratory tests show deviations of 1.78–5.26 dB when compared to professional devices like the Narda SRM-3006, although these are generally within acceptable ranges, they are still vulnerable to drift during prolonged outdoor use [25,26]. Frequency coverage gaps further hinder their ability to accurately represent conditions. Most low-cost sensors work within the Sub-6 GHz bands but cannot detect millimeter-wave (mmWave) emissions (24–100 GHz), which are becoming more important for urban 5G networks [57,58]. Although experimental integrated-circuit transceivers have shown they can operate cost-effectively up to 39 GHz [59], no open-source or consumer sensor provides the same stability or beam tracking capabilities at these higher frequencies. Ongoing challenges include the limits of dynamic range and detection thresholds. Budget devices typically saturate at around 20 V/m and lose sensitivity below 0.05 V/m. Environmental factors in 5G settings—such as beam switching, Time Division Duplexing (TDD), and Dynamic Spectrum Sharing (DSS)—further complicate measurements [60]. While standard averaging intervals, like 6 min ICNIRP windows, can obscure transient peaks, accurately capturing these rapid fluctuations often requires higher sampling rates and more precise synchronization than low-cost analogue-to-digital converters generally offer [27]. Empirical studies consistently quantify calibration drift and measurement uncertainty in practical deployments. For example, the Adalm-Pluto SDR platform exhibited drift of up to 3 dB after six months of outdoor use [25], while temperature variations of ±10 °C caused a 0.5–0.8 dB bias [53]. In contrast, the triaxial 5G sensor by Van der Straeten et al. [26] achieved stability within ±0.2 dB over 24 h, showing that environmental compensation circuits can significantly improve consistency. Most consumer-grade broadband meters (e.g., GQ EMF-390) report total measurement uncertainty of ±5–6 dB, roughly double that of professional equipment (±2–3 dB). These quantified differences underscore the necessity of periodic reference calibration and drift correction for long-term reliability in citizen and research deployments. Recent developments in related sensing fields indicate that machine learning-based calibration may help reduce systematic biases and environmental drift. Neural networks and random forest models have already increased the accuracy of low-cost air-quality sensors by 30–50% over linear models [61]. Applying such adaptive calibration techniques to RF-EMF monitoring—potentially integrating ambient temperature, humidity, and device orientation—represents an open yet promising area for future research.

### 5.2. Data Quality and Interpretation Obstacles

Apart from technical accuracy, the reliability of low-cost FR-EMF datasets is compromised by inconsistent methods and varying contexts. The absence of standardized measurement protocols for averaging, sampling frequency, and calibration leads to datasets that cannot be easily compared across different projects and countries [52]. Additionally, without standardized metadata or uncertainty reports, raw field data risks being misinterpreted or misused in public discussions. Furthermore, the variability of 5G emissions over time and space is significantly higher than that of legacy systems. Techniques like adaptive beamforming and traffic-based power adjustments lead to exposure levels that can fluctuate by several decibels within seconds or between neighboring micro-cells [25]. This variability complicates efforts to obtain accurate average exposure measurements from limited datasets. Another challenge is the gap between environmental field strength and personal exposure. While fixed or handheld sensors record ambient conditions, individual absorption varies greatly based on body orientation, closeness to devices, and shielding from clothing or surroundings. Studies with dosimeters reveal that personal exposure from one’s own mobile device can be one to two orders of magnitude higher than environmental measurements [29]. This disparity complicates epidemiological studies and public communication, as people often believe environmental readings directly indicate their personal dose. Standardizing data interpretation will thus need multi-modal methods that integrate affordable ambient sensors, personal dosimeters, and model-driven exposure assessments. Advances in spatial calibration techniques for sensor networks, like those used in air-quality monitoring, demonstrate that real-time bias adjustment and consistency across sites can be achieved with co-location learning models [62]. Such frameworks could also enhance citizen-driven RF-EMF networks.

### 5.3. Practical and Ethical Considerations

Deploying inexpensive sensors on a large scale also encounters practical and ethical challenges beyond measurement accuracy. Operational issues include unreliable power supply and connectivity; battery-powered sensors experience voltage drift that impacts sensitivity, while WiFi or LoRaWAN networks can cause latency and data losses [9,63,64,65]. Environmental factors such as heat, humidity, and rain reduce sensor durability, resulting in calibration issues or missing data [52]. The most pressing ethical concern is public misunderstanding. The bursty nature of 5G transmissions can lead to brief, high-amplitude peaks that exceed average exposure limits by several decibels, yet do not reflect genuine health risks. Without proper understanding, untrained users might interpret these raw, unaveraged peaks as hazardous, potentially spreading misinformation and leading to distrust [27]. Finally, privacy issues emerge due to the spatial granularity of dense sensor networks. Geolocated RF data can indirectly disclose device usage patterns, network congestion, or personal activities. Without explicit data-handling policies, community monitoring networks may breach privacy expectations or regulatory standards like the EU’s GDPR. Ethical governance in citizen air-quality projects highlights the importance of anonymization, data aggregation, and community consent to ensure sustainable participatory sensing [66]. The development of affordable 5G sensing technologies depends not only on technological advancements but also on upholding epistemic rigor and ethical responsibility. To produce credible, useful, and socially accepted data, a coordinated approach is necessary, combining precise technical calibration, standardized methods for data interpretation, and effective public communication strategies. Table 3 summarizes the main technical, interpretive, and ethical challenges encountered in low-cost RF-EMF sensing. Although measurement accuracy is still below professional levels, new approaches, such as machine learning calibration, colocation validation, and participatory governance, indicate a developing ecosystem that strives to balance precision with accessibility.

Table 3 highlights that developing affordable 5G RF-EMF sensing faces not only technical challenges but also epistemic and governance issues. Addressing calibration drift, protocol harmonization, and ethical data stewardship is crucial for these tools to transition from experimental prototypes to dependable additions to professional monitoring. The following section discusses the main research gaps in measurement science, data interoperability, and participatory governance that must be tackled to fully realize the potential of low-cost sensing for transparent and equitable 5G exposure assessment.

## 6. Identifying Critical Research Gaps

### 6.1. Metrology and Sensor Design

The future of affordable 5G RF-EMF sensing depends on developing strict metrological standards comparable to professional tools. Although prototype devices show promising accuracy with deviations as low as 1.78 to 2.8 dB when compared to laboratory-grade probes [25,26], there is still no standardized validation or calibration protocol tailored for 5G’s dynamic, beam-formed, and bursty signals. Currently, calibration methods rely on steady-state exposure in GTEM or anechoic chambers, which do not reflect real-world temporal modulation patterns. The field urgently needs calibration frameworks that address time-domain behavior, polarization diversity, and angular dependence specific to 5G New Radio (NR) emissions [27]. Expanding sensor frequency capabilities into the mmWave range (24–100 GHz) is equally urgent. Currently, low-cost sensors rarely go beyond 6 GHz, creating a gap in urban 5G deployments relying on FR2 bands. Recent advancements in low-cost intermediate-frequency transceivers built on 14 nm FinFET technology indicate that compact, flexible calibration systems for 39 GHz are now achievable [59,68]. The main engineering hurdle remains adapting these cost-effective innovations into mmWave isotropic probes for exposure monitoring. Another measurement challenge involves algorithmic error correction and vector fusion across different axes. While triaxial sensors are an advancement over single-axis devices, they still face issues like inter-axis coupling and inconsistent angular response. Using adaptive calibration matrices and machine-learning-based error fusion, similar to methods tested in low-cost air-quality sensors, could help reduce anisotropy and enhance isotropic accuracy [69]. Creating open, shareable calibration datasets and reference geometries would further foster a consensus in measurement standards.

### 6.2. Data Science and Modeling

While hardware advancements are essential, the current research gap lies in data fusion and modeling, which involves converting raw field strength data into scientifically valid and comparable exposure metrics. An immediate priority is combining low-cost data with high-quality professional measurements to improve the reliability of the entire network. Developing multilevel fusion frameworks that weight low-cost observations based on uncertainty or environmental factors is essential to avoid bias amplification when integrating heterogeneous data sources [70]. Another area of development involves creating algorithms that address angular dependence and variability in beamforming. Most low-cost sensors currently assume isotropic incident fields, leading to an underestimation of the anisotropic exposure patterns caused by 5G massive-MIMO and beam steering. Using simulation-based inverse modeling—integrating antenna radiation patterns with time-resolved power logs—could enable dynamic estimation of directional corrections [71,72]. Converting static measurements into realistic personal exposure metrics is still a complex challenge, both conceptually and computationally. Environmental readings indicate potential exposure but translating these into SAR or dose proxies involves modeling factors such as body posture, device proximity, and multipath signal propagation [29]. Combining ambient field maps with population mobility data could help generate probabilistic personal exposure estimates, which is an essential step for practical epidemiological applications. Finally, there is a lack of systematic uncertainty qualification pipelines. Few studies publish reproducible uncertainty budgets or use automated error propagation tools. Incorporating probabilistic modeling frameworks from air quality and radiation dosimetry could provide transparent uncertainty metrics for each observation. Additionally, advances in automated calibration methods, such as Random Forest and XGBoost models [69], should be applied to RF-EMF datasets. This would enable real-time bias correction and confidence estimation.

### 6.3. Implementation and Policy Frameworks

The process of achieving societal and regulatory integration depends on effective implementation logistics and governance structures. At present, there is no agreed-upon approach for maintaining, recalibrating, or auditing large-scale low-cost sensing networks over time. Sustained field deployments need plans for regular recalibration, energy independence, and data synchronization to maintain measurement accuracy. Regulator acceptance of citizen-sourced exposure data is still an emerging area. Although environmental agencies are gradually acknowledging the importance of participatory air-quality data, there is no similar policy framework for EMF exposure reporting. Developing reliable calibration methods, open metadata standards, and interoperable APIs could facilitate the incorporation of verified citizen datasets into national observatories. Ethical public communication requires careful design. Open dashboards, if not well-explained, may cause anxiety by highlighting short-term changes without averaging or uncertainty markers. Using risk communication principles (such as color scales matching ICNIRP thresholds and clear metadata) can improve understanding and prevent alarm. Citizen air-sensing network frameworks focus on transparency, obtaining consent, and ensuring data anonymization [66]. A commonly overlooked issue is the lack of standardized reporting practices. Presently, there is no unified template defining key calibration details, sampling intervals, averaging durations, or uncertainty estimates for low-cost EMF sensors. Creating such standards, inspired by FAIR (Findable, Accessible, Interoperable, Reusable) data principles, would greatly improve reproducibility and support regulatory acceptance. Recent European research initiatives have advanced the standardization of RF-EMF exposure monitoring, especially under 5G conditions. The SEAWAVE (Systematic Review on EMF Exposure and 5G) project, supported by Horizon Europe, systematically reviews exposure metrics, biological effects, and epidemiological evidence related to 5G, aiding in the improvement of health risk assessment methods [73,74,75]. Additionally, the GOLIAT (Global System for Mobile Communication Exposure Assessment and Epidemiology) project develops harmonized exposure models, personal monitoring protocols, and cross-national epidemiological frameworks to connect population-level and environmental measurements [76,77]. These efforts lay an essential groundwork for incorporating participatory and low-cost sensing systems into scientifically sound and policy-relevant exposure assessments across Europe.

## 7. Discussion and Future Directions

### 7.1. Technical Context of 5G and Wi-Fi Standards in Exposure Monitoring

The studies analyzed indicate that affordable RF-EMF sensors need to accommodate the spectral and signaling characteristics of both 5G New Radio (NR) and WiFi (IEEE 802.11) technologies. 5G NR operates across two frequency ranges: Frequency Range 1 (FR1, 0.7–6 GHz) and Frequency Range 2 (FR2, 24–100 GHz), utilizing advanced features like massive-MIMO beamforming, dynamic spectrum sharing, time-division duplexing (TDD), and carrier aggregation. Environmental exposures in FR1 typically range from 0.01 to 0.5 W/m^2^, with localized peaks reaching up to 2 W/m^2^ near small cells [9,25]. Wi-Fi technologies (Wi-Fi 4 at 2.4/5 GHz, Wi-Fi 5 at 5 GHz, Wi-Fi 6/6E spanning 2.4–7 GHz, and HaLow at 0.9 GHz) emit intermittently, usually with duty cycles below 10%, resulting in highly variable instantaneous field strengths but relatively low average exposures [29,40]. Research indicates that low-cost Sub-6 GHz sensors can effectively monitor both 5G FR1 and Wi-Fi 4–6E, achieving accuracy levels of ±3–6 dB. However, measurements at mmWave frequencies (FR2 above 24 GHz and WiFi 7/8 around 60 GHz) are still constrained by antenna design and component costs [26,27]. The most successful sensor setups combine triaxial isotropic antennas, SDR-based signal processing, and adaptive calibration to adapt to the dynamic temporal and spectral features of these standards. Incorporating this understanding of radio interference characteristics ensures that exposure assessments remain technically rigorous and that the discussion of results accurately reflects the operational realities of current wireless systems.

### 7.2. An Interdisciplinary Agenda

The future of low-cost 5G RF-EMF sensing relies on strong interdisciplinary collaboration. Engineers, data scientists, epidemiologists, and regulators each contribute a crucial part to the exposure-monitoring puzzle. Engineers are pushing hardware limits by creating triaxial isotropic sensors and mmWave-capable probes with less than 3 dB uncertainty [26,68]. Meanwhile, data scientists are designing machine learning models to correct biases, fill spatial gaps, and reconstruct transient 5G beam patterns [78]. Epidemiologists and exposure scientists need to work together to convert raw sensor data into metrics that are relevant for dose assessment, closing the gap between environmental measurements and individual exposure estimates [29]. Regulators and standards organizations (such as ICNIRP, IEC, and CENELEC) must then verify that participatory data align with safety limits and uncertainty considerations. Interdisciplinary collaboration will determine whether low-cost sensing can become a reliable supplement to official monitoring or if it remains a scattered citizen science effort lacking policy influence.

### 7.3. Differentiated Applications

The value of low-cost RF-EMF sensing depends on defining its use cases, as various applications require different performance standards [25]. For monitoring regulatory compliance, accuracy and traceability are essential. Devices need calibration traceable to national metrology standards and must maintain measurement uncertainty within ±2–3 dB [79]. For public engagement and risk communication, transparency, accessibility, and interpretability are more important than absolute accuracy [80]. Community dashboards, like those tested in Greece’s National Observatory of EMF Exposure, present promising models for participatory data sharing [18], as long as they clearly show contextual uncertainty and averaging windows. In health research, ensuring data reliability, temporal detail, and contextual metadata is crucial. Longitudinal exposure studies should use harmonized field strength measurements with consistent averaging intervals (e.g., 6 min ICNIRP standards) and incorporate robust methods for uncertainty propagation [27]. By differentiating these application layers—compliance, communication, and research—the field can better align sensor design, calibration depth, and governance requirements.

### 7.4. Case Study Vignettes

Recent European initiatives demonstrate both the potential and constraints of affordable 5G sensing. In Belgium and the Netherlands, the Smart Sensor Systems (S^3^R) network illustrates a hybrid citizen-academic approach, integrating affordable nodes with official oversight. These networks deliver spatial resolutions that surpass those of traditional campaigns, while deviations stay below 5 dB compared to certified instruments [25]. Greece’s EMF Observatory at the national level combines data from both citizens and professionals in public dashboards, supported by calibration campaigns led by universities. Its success is due to a two-tier validation process, where citizen-collected data is cross-checked with data from certified sensors [18]. Laboratory benchmarking continues to validate field results. In controlled comparisons between the SRM-3006 professional meter and the triaxial low-cost 5G sensor by Ven der Straeten et. al, the triaxial low-cost 5G sensor showed deviations of only 2.8 dB, which falls within the commercial system’s uncertainty range [26]. These findings demonstrate that, with thorough calibration and quality control, affordable sensors can effectively supplement professional infrastructure both scientifically and socially. Qualitatively, these systems exhibit deviations of 2 to 5 dB from reference instruments and long-term drift rates under 0.5 dB per month. This confirms that modern low-cost sensors nearly match professional stability in real-world deployment.

In addition to the European citizen sensing initiatives discussed earlier, international research teams have achieved significant progress in high-resolution modeling of RF-EMF power density distributions. Manassas et al. [81] developed a machine learning framework that combines measurement data and GIS parameters to predict electromagnetic field exposure in complex urban environments, demonstrating how data-driven modeling can improve spatial estimation.

Similarly, Salem et al. [82] employed 3D ray-tracing and beamforming simulations to analyze uplink and downlink exposure in dense urban 5G networks, emphasizing the significant spatial variability in predicted power density and SAR across city environments. Ramirez-Vazquez et al. [41] conducted a systematic review and comparison of spot-measurement and mixed-method studies, illustrating how combined fixed and mobile measurements can effectively capture the variability of real-world RF exposure patterns. Moreover, comprehensive measurement-based assessments [83] using distributed drive-test networks have shown how combining stationary sensors with mobile surveys can produce detailed spatial maps of 5G downlink emissions. These high-precision modeling efforts deliver critical baseline data for understanding exposure heterogeneity but remain resource-intensive and are generally confined to research or regulatory contexts. In contrast, low-cost and participatory monitoring systems can provide complementary, distributed, and continuously updated exposure data at minimal cost, fostering transparency and community engagement.

Recent studies, including Najera et al. [28], show that combining personal dosimeters with static low-cost sensor nodes produces more accurate and stable EMF exposure maps over time than using either method alone. Their use of evolutionary programming to optimize sensor placement and calibration highlights a promising direction in low-cost sensing research. Incorporating such computational techniques into participatory networks could significantly improve spatial resolution and scientific reliability. This trend indicates that low-cost sensing is advancing from conceptual ideas to validated hybrid systems, supporting the practical potential discussed throughout the review. This synergy between top-down modeling and bottom-up sensing forms the methodological bridge this review aims to emphasize, highlighting the importance of integrating democratized data collection with advanced scientific modeling to achieve comprehensive 5G exposure assessment.

### 7.5. Standardization and Interoperability

The most significant factor for long-term impact is standardization and interoperability. At present, low-cost sensing exists within a fragmented ecosystem, with each research group using unique sampling frequencies, calibration protocols, and metadata schemas [27]. An international effort similar to CONSORT (for clinical trials) or PRISMA (for systematic reviews) could establish minimal reporting standards, including the calibration chain, frequency coverage, averaging interval, uncertainty budget, and data format. Without such frameworks, cross-study comparability and regulatory integration will remain limited. The efforts by the European Telecommunication Standards Institute (ETSI) and IEEE EMC committees already indicate a move toward standardizing RF-EMF measurement procedures for 5G small-cell environments [84]. However, extending these standards to low-cost, distributed networks will necessitate open metadata schemas and machine-readable calibration certificates. Insights from the FAIR data principles and citizen science codes of conduct highlight that transparency, traceability, and interoperability should be integrated from the start, rather than as afterthoughts.

## 8. Conclusions

The review examined over 60 studies on low-cost 5G and Wi-Fi RF-EMF exposure sensors, showing that most Sub-6 GHz systems have measurement deviations of ±3–6 dB compared to certified reference meters. Long-term outdoor deployments report calibration drift of less than 0.5 dB per month, while indoor tests show reproducibility within 5% RMs error. Typical ambient 5G FR1 exposure levels ranged from 0.01 to 0.5 W/m^2^, rising to about 2 W/m^2^ near small cells. Personal handset use can cause transient exposures 10 to 30 dB higher. These data demonstrate that affordable sensors, when properly calibrated, can approximate professional accuracy for community monitoring of 5G and Wi-Fi 4–6e fields. Nevertheless, measurement challenges persist for FR2 frequencies above 24 GHz and Wi-Fi 7/8 (60 GHz) due to antenna and component limitations. Future efforts should focus on mmWave calibration, standardized uncertainty assessment, and data-integration protocols to support transparent, large-scale exposure mapping.

## Figures and Tables

**Table 1:** Comparison of RF-EMF Exposure Assessment Approaches.

Monitoring Approach	Typical Instruments/Methods	Accuracy and Calibration	Spatial/Temporal Coverage	Cost and Accessibility	Data Transparency and Public Engagement	Key Limitations	Sources
Professional Monitoring (regulatory/Research)	Isotropic E-field probes, spectrum analyzers, frequency-selective meters, dosimeters; drive-by surveys, stationary monitoring networks	High—laboratory-calibrated instruments with traceable standards	Limited spatially, high temporal precision (in fixed locations)	High cost, specialized expertise required	Low—data often aggregated or delayed; limited public access	Sparse geographic coverage; episodic campaigns; inaccessible raw data	[18,40,41]
Low-Cost Sensor Networks (Research/Institutional Pilot Projects)	Compact RF power sensors, SDR-based detectors, IoT integrated sensor nodes	Moderate—calibration against reference instruments required; sensitivity varies	High spatial, continuous temporal coverage possible	Low to moderate cost, scalable deployments	Moderate—public dashboards increasingly used (e.g., NOEF Greece, France ANFR trials)	Calibration drift, signal discrimination challenges, environmental noise	[27,42]
Participatory Citizen Science Monitoring	Low-cost handheld EMF meters, smartphone-based detectors, community sensor kits	Variability—depends on sensor type, calibration, user training	Very high spatial, temporal (depends on volunteer activity)	Low cost, open participation	High—fosters trust, data sharing, cocreation of knowledge	Limited accuracy; inconsistent protocols; potential data misuse	[27,29,41]

**Table 2:** Representative Low-Cost RF-EMF Sensors and Their Technical Characteristics. The table lists selected low-cost and research-grade sensors, along with one professional reference instrument (Narda SRM-3006, Pfullingen, Germany) included for comparative benchmarking.

Device/Platform	Approx. Cost (EUR)	Frequency Range (GHz)	Dynamic Range	Measurement Type	Claimed Accuracy/Uncertainty	Key Features and Notes	References
* Narda SRM-3006 (Professional Reference Instrument)	>10,000	0.1–40 (depends on probe)	~100 (manufacturer spec)	Frequency-selective isotropic probe	±1.5 dB (confirmed in metrology calibration)	Laboratory-calibrated reference instrument used by research institutions, regulators and for ICNIRP compliance; high portability; real-time spectral analysis	[44,45,46,47,48]
Adalm-Pluto SDR Analog Devices, Figure 1b)	~400	0.325–3.8	0.1–20	Broadband/SDR-based	±4–6 dB	Open source SDR platform frequently adapted in research for 5G Sub-6 GHz sensing	[25]
ExposureSure Node v3 (IoT sensor)	~800	0.7–6.0	0.2–30	Broadband/IoT node	±3 dB	Cloud-connected EMF sensor designed for distributed network deployment; limited frequency selectivity	[27]
GQ EMF-390 (Consumer Handheld, Figure 1a)	~300	0.1–8.0	0.1–20	Broadband handheld	±5–6 dB	Widely available consumer meter combining EMF, ELF, and RF modes; coarse spectral discrimination	Manufacturer specifications
Low-Cost Triaxial 5G Sensor (Research Prototype)	~1000–1200	3.3–4.2 (5G n77/n78)	0.06–30	Triaxial analog-to-digital design	±3.12 dB	Field-validated against SRM-30006; deviation~2.8 dB within reference uncertainty	[26]

* The Narda SRM-3006 is not a low-cost device but is included as a professional benchmark for comparison with affordable and prototype sensors.

**Table 3:** Summary of Core Challenges in Low-Cost 5G RF-EMF Sensing and Emerging Mitigation Strategies.

Challenge Domain	Key Issues	Typical Impact	Emerging Mitigation Strategies	Reference
Metrological and technical aspects	Calibration drift, frequency coverage gaps (especially mmWave); limited dynamic range; inability to capture transient 5G signals (beamforming, DSS)	Measurement uncertainty ±3–6 dB; field calibration drift ~0.3–0.5 dB/month; temperature bias up to 0.8 dB.	Periodic co-location reference probes; temperature-compensated circuits; ML-based calibration and drift correction	[9,25,26,53,67]
Data quality and interpretation	Lack of harmonized measurement protocols; inconsistent metadata; temporal variability; mismatch between ambient and personal exposure.	Non-comparable datasets; risk of public misinterpretation; limited integration into official systems.	Standardized metadata templates; multi-modal sensing (ambient + personal); spatial co-location and bias correction frameworks.	[29,52,59]
Practical and ethical considerations	Power supply and network instability; weather-related degradation; risk of public alarm from transient peaks; privacy and geolocation concerns.	Data loss; public distrust; potential privacy violations.	Durable enclosures and energy harvesting; automated time-averaging and filtering; anonymization and aggregation data publishing.	[27,66]

## Data Availability

Not applicable.
